# Heart rate changes during electroconvulsive therapy

**DOI:** 10.1186/1744-859X-12-19

**Published:** 2013-06-13

**Authors:** Josef Nagler

**Affiliations:** 1 Christophsbad Clinic, Göppingen 73035, Germany; 2 Center of Psychiatry, Winnenden 71364, Germany

**Keywords:** Electroconvulsive therapy, Heart rate, Asystole, Bifrontal stimulation, Baroreceptor reflex

## Abstract

**Background:**

This observational study documented heart rate over the entire course of electrically induced seizures and aimed to evaluate the effects of stimulus electrode placement, patients' age, stimulus dose, and additional predictors.

**Method:**

In 119 consecutive patients with 64 right unilateral (RUL) and 55 bifrontal (BF) electroconvulsive treatments, heart rate graphs based on beat-to-beat measurements were plotted up to durations of 130 s.

**Results:**

In RUL stimulation, the initial drop in heart rate lasted for 12.5 ± 2.6 s (mean ± standard deviation). This depended on stimulus train duration, age, and baseline heart rate. In seizures induced with BF electrode placement, a sympathetic response was observed within the first few seconds of the stimulation phase (median 3.5 s). This was also the case with subconvulsive stimulations. The mean peak heart rate in all 119 treatments amounted to 135 ± 20 bpm and depended on baseline heart rate and seizure duration; electrode placement, charge dose, and age were insignificant in regression analysis. A marked decline in heart rate in connection with seizure cessation occurred in 71% of treatments.

**Conclusions:**

A significant independent effect of stimulus electrode positioning on cardiac action was evident only in the initial phase of the seizures. Electrical stimulation rather than the seizure causes the initial heart rate increase in BF treatments. The data reveal no rationale for setting the stimulus doses as a function of intraictal peak heart rates (‘benchmark method’). The marked decline in heart rate at the end of most seizures is probably mediated by a baroreceptor reflex.

## Background

Cardiac action in electrically induced seizures follows a characteristic phase-dependent course with rapid changes [1]. In cases of unilateral or bitemporal electrode positioning, the administration of electrical stimuli is accompanied by temporary asystole. The succeeding clonic phase of the seizure is marked by tachycardia, arrhythmia, and hypertension. Finally, the cessation of clonic movements sometimes coincides with another slowing of the heart rate (HR) [2,3] whereas blood pressure continues to be elevated for a few minutes longer [4].

The measurement of pulse rate and blood pressure once a minute appears to be inadequate for investigating such rapidly changing cardiac action. Besides, automated non-invasive blood pressure monitoring takes some time, often more than 30 s, and cardiac action may not remain constant during this period. In view of the rapid changes that take place, precise statements regarding intraictal blood pressure values require arterial catheterization [3] or a special beat-to-beat monitoring device [5]. The accurate measurement of heart rate is, in comparison, easy. Recording of electrocardiogram (ECG) and pulse oximeter curves is appropriate and well established in the setting of anesthesia and convulsive therapy. Despite that easy accessibility, studies on a beat-to-beat basis have rarely been conducted, and their results are conflicting with regard to the effects of age and stimulus dose.

Huang et al. [6] investigated the magnitude of cardiovascular changes in 13 consecutive patients. The maximal heart rate during the first treatment session had a mean of 143 ± 25 beats per minute (bpm). It fell significantly with age, and there was no association between energy used and hemodynamic response. Swartz and Shen [7] reported on a large non-consecutive sample of 177 patients. The majority had left-anterior right-temporal electrode placement, and 40 patients received bifrontotemporal placement. Regression analysis showed a small but significant decline of peak HR with age. After separating the data points into two clusters, the authors stated a desirable peak HR of 140 to 180 bpm, regardless of age.

In another paper [8], Swartz reported on heart rate responses following stimulation with half-age dose and age-based dose. Electrode placement was in the left-anterior right-temporal position. This study comprised 24 consecutive patients. It included five patients with absent or inadequate seizures in the group with half-age dosing. Their peak heart rates were evidently lower than during a fully developed seizure. By retaining four of these patients in his data analyses, the author was able to demonstrate significantly different heart rates between the dosing regimes. A one-tailed *t* test after removal of these subjects resulted in *p* = 0.044, so an ordinary two-tailed *t* test would have shown no impact of the stimulus dose on the peak heart rates in regular seizures.

The following observational study describes the cardiac action in the course of 119 seizures induced by right unilateral (RUL) or bifrontal (BF) stimulation. In the same patient sample, a previous analysis of vagal activation during the stimulation period [9] revealed a mean asystole duration of 5.64 ± 2.88 s in RUL electroconvulsive therapy (ECT). The application of electrical pulses via BF electrodes was not associated with cardiac pauses. In the present study, we evaluated whether the subsequent intraictal cardiac action was different between RUL and BF ECT. The data pool was also suitable for re-assessing the impact of age and stimulus dose on heart rate.

## Methods

Data were collected prospectively from all patients undergoing electroconvulsive therapy in two hospitals between October 2008 and June 2010. A total of 119 patients received treatment series with either RUL (*n* = 64) or BF (*n* = 55) stimulation. Both clinics made use of a Thymatron II device (Somatics Inc., Lake Bluff, IL, USA) which provided a maximum charge of 1,008 mC with current held constant at 0.9 A. For RUL stimulation, an adhesive electrode was affixed to the right temple, and the second electrode (mounted on a handle) was placed adjacent to the vertex. For BF stimulation, two adhesive electrodes were applied above the lateral halves of the eyebrows, the alignment commonly being vertical. The distance between their midpoints varied from 8.4 to 12 cm and averaged 10.3 ± 0.7 cm. The selection of the electrode placement and the setting of the stimulus dose were at the discretion of the attending psychiatrists. Concomitant medications remained unchanged with the exception of the dose before anesthesia. Further details on treatment procedures and patient characteristics have been described previously [9].

Anesthesia started with the IV administration of 0.25 mg atropine. Sleep was induced with etomidate, 0.2 mg/kg, followed by succinylcholine at a dose of 100 mg in the first session. No additional drugs were administered in the routine procedure, except for propofol to terminate seizures lasting longer than 80 s. All patients were ventilated using a face mask, so that an oxygen saturation of 100% and an end-tidal pCO_2_ ≤ 35 mmHg were attained before stimulation. Anesthesia monitoring comprised ECG, pulse oximetry, capnometry, and non-invasive blood pressure. During the actual convulsive treatments, data were also recorded on paper printouts. The same anesthetist was engaged in both clinics throughout the study period.

### Measurements and statistics

**Figure 1:**
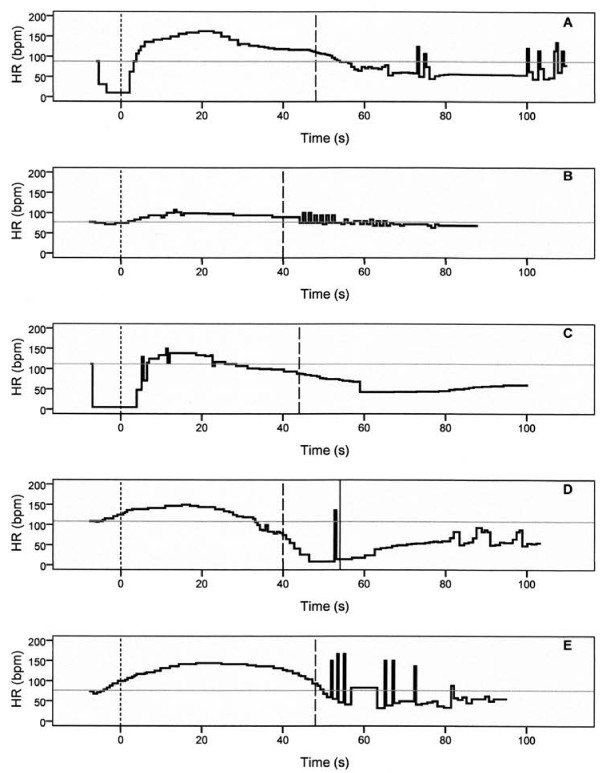
**Heart rate curves during electroconvulsive therapy.** Horizontal gray lines, baseline heart rate; dotted vertical lines, end of stimulation; broken vertical lines, end of motor seizure. (**A**) 39-year-old patient; RUL treatment, 151 mC; stimulus duration, 5.6 s. Baseline HR, 83 bpm; peak HR, 163 bpm; *T*_rise_, 9.3 s; *T*_max_, 19.2 s; *T*_base_, 54.0 s; *T*_dec_, 58.5 s; motor seizure duration, 48 s. Gradual decline in heart rate at 25 s indicating either mitigation of sympathetic impact or activation of parasympathetic forces during the seizure and a second decline following the convulsive period. (**B**) 68-year-old patient; RUL stimulation, 195 mC, 7.5 s. Heart rate changed moderately from 77 to 107 bpm, and an abrupt decline at the end of the seizure was absent. Medical evaluation revealed an impaired atrioventricular conduction combined with a bundle branch block. (**C**) 43-year-old patient; RUL stimulation, 203 mC, 7.5 s. Asystole of 10.9 s duration; *T*_rise_, 13.9 s; motor seizure duration, 44 s. The electrode placement was switched to BF position in this patient to prevent cardiac standstill during stimulation. Doses and treatment technique remained otherwise unchanged (**D**). *T*_rise_, 2.9 s; motor seizure duration, 40 s; EEG seizure duration, 54 s. The post-ictal heart rate slowdown proceeded to an asystole of 6.2 s duration. Medical evaluation was unremarkable, and pharmacotherapy consisted of citalopram. (**E**) 76-year-old patient; BF treatment, 301 mC, 6.7 s. *T*_rise_, 2.6 s. The seizure end at 48 s was accompanied by a steep decline; the succeeding period of vagal dominance was marked by arrhythmia.

All R-R intervals in the ECG printout were measured manually with a ruler and transformed into seconds. Their cumulative sum forms the horizontal time axis for the heart rate graphs shown in Figure 1. The heart rate depiction on the vertical axis is based on the single R-R intervals. The pulse oximeter curve served as a substitute in case of impaired ECG recordings during the stimulation phase. To keep the effort within limits, the evaluation covered a maximum period of 130 s (including stimulation) and was confined to the first treatment session of each patient.

If the first treatment in an individual series could not be evaluated, the subsequent treatment was analyzed. As seizure durations commonly decrease during a treatment series, that subsequent seizure was expected to be shorter than the actual first one of the series. The resulting bias was determined by re-analyzing the sample without those patients.

To characterize the heart rate graph, a total of ten outcome variables were defined. The maximum heart rate in the course of the seizure (peak HR) was calculated from the shortest R-R interval in the ECG record; ectopic beats were excluded. Further outcomes were the time from the onset of stimulation until the heart rate rose above baseline level (*T*_rise_) and the time to peak heart rate (*T*_max_). Tachycardia duration was assessed in two ways: (1) the time period from the end of stimulation until the heart rate fell to its baseline level again (*T*_base_) and (2) the time to the center of the sharpest heart rate decline within a 5-s period (*T*_dec_) if a pronounced decline was present. With the exception of *T*_rise_, time (s) was always measured from the end of stimulation. The speed of decline in heart rate at the end of the seizure was calculated by HR at the end of the seizure minus HR 20 s later. Final HR denoted the heart rate before transfer to the post-anesthesia care unit. Motor seizure duration was determined in the right foot, which had been cuffed prior to the injection of succinylcholine. Electroencephalogram (EEG) seizure duration was taken into account only if a flatline was visible within 2 s after unequivocal seizure signs, in accordance with a suppression index >90%. The term seizure duration refers to motor seizure duration unless otherwise stated.

This was an observational study, and as in all such investigations, bias could be introduced by imbalances in baseline characteristics. Bivariate comparisons were, therefore, considered less conclusive and were reported only for descriptive reasons. The independent effects of age, charge dose, and electrode positioning (RUL versus BF) on outcomes of interest were assessed in multiple regression analysis. If clinical experience or previous investigations suggested further predictors, they were added in the process of model building. SPSS 16.0 was used for the calculations, and all significance tests were two-sided. A total of eight regression analyses were carried out. To compensate for multiple testing, the level of significance for the regression coefficients was adjusted to *p* = 0.01. Because the preceding bivariate correlations were considered to be descriptive, no adjustments were made for multiple comparisons in these analyses. The corresponding *p* values should be interpreted accordingly.

## Results

Upon arrival in the ECT unit, the patients, still aware and oriented, had a mean heart rate of 82 ± 14 bpm and a mean blood pressure of 136 ± 21 / 74 ± 11 mmHg. The mean heart rate in anesthetized patients immediately before administration of the electrical stimuli was 90 ± 18 bpm. Automated blood pressure was monitored once a minute; however, owing to divergent measuring times, no clear assignments to seizure time points could be established. The diastolic level in definite intraictal measurements regularly exceeded 100 mmHg. When the patients were transferred to the post-anesthesia care unit, their mean heart rate was again 81 ± 15 bpm, and the average blood pressure was 130 ± 22 / 72 ± 15 mmHg.

Table 1 shows the baseline characteristics of the patients according to electrode positioning (RUL versus BF). The attending psychiatrists preferred bifrontal electrode placement in the elderly and stimulus doses that increased with age. Thus, age and charge dose were unevenly distributed among the groups. However, given the paucity of data in this field, all outcome variables, separately for RUL and BF treatments, are listed in Table 2 as raw data, with significance tests unadjusted for covariates. The independent effects of the predictors, corrected for baseline imbalances, were subsequently ascertained by multiple regression analysis. The results are reported below in the order of the outcome variables.

**Table 1:** Baseline characteristics in right unilateral and bifrontal treatments

	**RUL ECT (*n *= 64)**		**BF ECT (*n *= 55)**		***p *value**
	**Mean ± SD**	**Median (range)**	**Mean ± SD**	**Median (range)**	
Age (yr)	53 ± 12	52 (29–79)	71 ± 7	71 (43–86)	0.000^a^
First HR (bpm)	81 ± 13	80 (56–123)	83 ± 15	83 (50–124)	0.490^a^
Baseline HR (bpm)	90 ± 17	88 (55–127)	89 ± 18	88 (60–139)	0.677^a^
Charge dose (mC)	222 ± 96	197 (88–605)	350 ± 174	323 (151–1014)	0.000^b^

SD, standard deviation; range, minimum-maximum range; First HR (bpm), heart rate (beats per minute) upon arrival in the ECT unit; Baseline HR (bpm), heart rate (beats per minute) immediately before stimulation; ^a^*t* test; ^b^Mann–Whitney *U* test.

**Table 2:** Outcome variables in right unilateral and bifrontal treatments

	**RUL ECT (*n *= 64)**		**BF ECT (*n *= 55)**		***p *value**
	**Mean ± SD**	**Median (range)**	**Mean ± SD**	**Median (range)**	
Peak HR (bpm)	137 ± 20	136 (88–187)	133 ± 20	134 (91–167)	0.296^a^
*T*_rise_ (s)	12.5 ± 2.6	12.5 (6.3–17.8)	4.5 ± 3.1	3.5 (0.7–14.6)	0.000^b^
*T*_max_ (s)	20.2 ± 8.8	19.1 (9.1–61.2)	21.3 ± 10.4	19.4 (0.0–60.1)	0.535^b^
*T*_base_ (s)	55.8 ± 21.7	54.0 (13.3–107.2)	67.0 ± 25.9	62.1 (21.2–117.7)	0.026^a^
*T*_dec_ (s)	54.3 ± 21.9	54.7 (13.3–107.6)	62.1 ± 18.7	61.0 (22.1–95.8)	0.063^b^
HR at seizure end (bpm)	114 ± 27	115 (45–163)	119 ± 23	121 (50–163)	0.271^a^
HR 20 s after seizure (bpm)	78 ± 27	71 (28–156)	94 ± 22	96 (50–139)	0.002^b^
Final HR (bpm)	81 ± 14	81 (50–115)	82 ± 18	80 (49–125)	0.742^a^
Seizure duration (s)	45 ± 12	45 (13–78)	51 ± 13	51 (28–85)	0.013^a^
EEG seizure duration (s)	74 ± 26	70 (19–116)	55 ± 15	54 (33–83)	0.057^a^

SD, standard deviation; range, minimum-maximum range; HR (bpm), heart rate (beats per minute). Time (s) is always measured from the end of stimulation with the exception of *T*_rise_.

*T*_rise_, time from the onset of stimulation until the heart rate rises above baseline level; *T*_max_, time to peak heart rate; *T*_base_, time until the heart rate falls to baseline level; *T*_dec_, time to the steepest heart rate decline during and after the seizure; Final HR, heart rate before transfer to the post-anesthesia care unit. ^a^*t* test; ^b^Mann–Whitney *U* test.

The mean peak HR in all treatments was 135 ± 20 bpm. Bivariate analyses showed a moderate correlation with baseline HR (*r* = 0.358, *p* = 0.000) and another one with the patients' age (*r* = −0.190, *p* = 0.041). Charge dose had no effect on the peak HR (*r* = −0.135, *p* = 0.149). The same applied to the positioning of the stimulus electrodes (Table 2, *p* = 0.296). The time taken to reach the peak HR (*T*_max_) overlapped the seizure durations (Table 2), and an association between tachycardia and seizure duration was common, leading to the assumption of lower peak HRs in short seizures. There was in fact some association between motor seizure duration and peak HR (*r* = 0.195, *p* = 0.038). The multiple regression analysis confirmed the baseline HR (*B* = 0.456, *p* = 0.000) and seizure duration (*B* = 0.396, *p* = 0.005) as independent predictors. A higher baseline HR of 10 bpm was associated with a higher peak HR of 5 bpm and reducing seizure durations by 10 s brought down peak HRs by 4 bpm on average.

The time until the HR started rising above baseline (*T*_rise_) was considerably longer in RUL ECT than in BF stimulation and showed a bimodal distribution with a median of 3.5 s in BF electrode placement and 12.5 s in RUL positioning. As evident from Table 3, the 9 s earlier increase in heart rate during BF stimulation was independent of age, charge dose, and baseline HR.

**Table 3:** Regressions of four outcome variables (row 1) on assumed predictors (column 1)

	**Peak HR**	***T***_**rise**_	***T***_**rise **_**RUL**	***T***_**dec**_
Constant	91.541 (15.808)*	1.525 (1.771)	−4.844 (2.250)	6.042 (11.994)
Age (yr)	−0.193 (0.176)	0.084 (0.023)*	0.075 (0.022)*	0.039 (0.178)
Charge dose (mC)	−0.015 (0.013)	0.002 (0.002)	−0.003 (0.003)	−0.014 (0.013)
Positioning^a^	0.402 (4.810)	−9.630 (0.621)*		2.602 (5.376)
Baseline HR (bpm)	0.456 (0.101)*	0.068 (0.013)*	0.061 (0.014)*	
Stimulus duration (s)			1.273 (0.266)*	
Seizure duration (s)	0.396 (0.139)*			1.093 (0.139)*
Number	114	111	63	84
Adjusted *R*^2^	0.195	0.756	0.510	0.460
*F*	6.465	86.108	17.161	18.710

*T*_rise_, time from the onset of stimulation until the heart rate rises above baseline level; *T*_rise_ RUL, *T*_rise_ in patients with right unilateral stimulation; *T*_dec_, time to the steepest heart rate decline during and after the seizure. Cell values in predictor rows are *B* coefficients with standard errors in parentheses; *p values < 0.01. ^a^Relative to RUL positioning.

The onset of tachycardia was traceable in half of the BF treatments as early as 3.5 s after initiation of the electrical pulses. At the end of the stimulation period, which lasted for 7.2 s on average, a higher heart rate was present in 43 of the 55 patients. None of the predictors listed in Table 3 had an influence on *T*_rise_ in the BF subgroup after an outlier (baseline HR 139 bpm, *T*_rise_ = 14.6 s) was removed.

In RUL treatments, bivariate analyses revealed correlations of *T*_rise_ with age (*r* = 0.400, *p* = 0.001), stimulus duration (*r* = 0.574, *p* = 0.000), and baseline HR (*r* = 0.407, *p* = 0.001). The predictors remained significant in regression analysis. It took longer for older patients to exceed their baseline level, and it took more time to exceed a higher baseline. Clinical relevance can be attributed to the high coefficient of the stimulus duration (*B* = 1.27, *p* = 0.000). Extending the duration by 1 s prolonged the corresponding parasympathetic phase by 1.27 s.

The time interval between stimulation and peak HR (*T*_max_) did not differ in RUL and BF ECT. Regression analysis established an effect of age (*B* = 0.356, *p* = 0.000) and frequency (*B* = −0.201, *p* = 0.006). The calculation, however, was flawed by heteroscedasticity and its predictive value was only 12%.

At the end of the seizures, the cardiac rate dropped below baseline in 93 patients (78%). Short self-limiting asystole events could be seen in RUL as well as in BF treatments. The longest pause in the 119 ECGs of this sample lasted for 4.1 s. In 26 patients, the heart rate remained above baseline or could not be determined because of frequent extrasystoles. The time at which the heart rate fell to its baseline level (*T*_base_) was often associated with the termination of motor convulsions (*r* = 0.620, *p* = 0.000). There were additional correlations with baseline HR (*r* = −0.217, *p* = 0.036) and peak HR (*r* = 0.276, *p* = 0.007), but they were trivial if *T*_base_ was chosen as the outcome variable.

Taking the point of greatest heart rate deceleration (*T*_dec_) as the end point of tachycardia resulted in a similarly high correlation with the convulsive period (*r* = 0.692, *p* = 0.000). As shown in Table 3, there were no confounding variables. The effects of baseline and peak HR were likewise negligible. A formal correlation existed with EEG seizure duration (*r* = 0.774, *p* = 0.000). In most cases, however, *T*_dec_ preceded the EEG end point, so the latter variable did not seem to be a suitable predictor for the former. An abrupt EEG end point was only recorded in 25 RUL and 8 BF treatments.

The speed of decline in heart rate at the end of the seizure could be assessed in 94 patients. In the 20 s following the termination of seizure activity, the heart rate fell by 30 ± 20 bpm with a median of 25 and a range of −3 to 89 bpm. The decline was faster in the case of higher peak HRs (*B* = 0.384, *p* = 0.000); it was attenuated in lengthy seizures (*B* = −0.434, *p* = 0.003) and in older patients (*B* = −0.315, *p* = 0.027).

No conclusions could be drawn regarding the determinants of the seizure duration. There were only weak correlations indicating prolonged seizures after BF stimulation (Table 2) and, in the RUL subgroup, a longer convulsive period in younger patients and those with a lower charge dose. A valid regression equation could not be established. The records of the first treatment were incomplete in 11 patients (9%), and we thus evaluated a subsequent session instead. This could have biased the calculations referring to seizure durations. However, when excluding these patients from the analysis, mean seizure durations proved to be only 1 s longer in RUL and BF treatments, and the pattern of significance in the equations of Table 3 remained unchanged.

## Discussion

### Peak heart rate

In our sample of 119 patients, peak heart rates during electroconvulsive therapy ranged from 88 to 187 bpm and averaged 135 ± 20 bpm. Only 50 patients exceeded a heart rate of 140 bpm during seizure. The corresponding mean in the study of Swartz and Shen [7] was 154 ± 22 bpm for men and 160 ± 17 bpm for women. The higher numbers might be due to their selection of the peak heart rate of each patient from all treatments. Huang et al. and the present study focused on the first session only. Furthermore, heart rate numbers on a digital display, as used by Swartz and Shen, might include ectopic beats and additional interference.

In line with Huang et al. [6] and Swartz and Shen [7], we found an effect of age on peak HR in bivariate analysis. However, it was small and lost its significance when baseline HR and seizure duration were taken into account.

**Figure 2:**
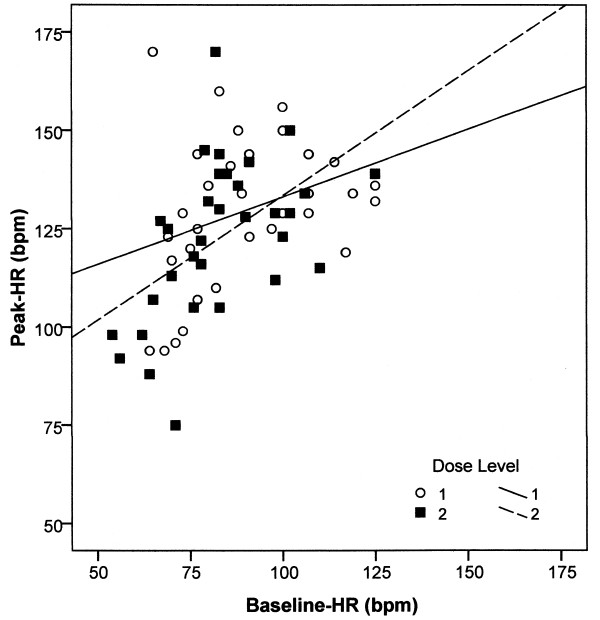
**Peak heart rate as a function of baseline heart rate and charge dose.** In 32 patients, the initial charge dose (level 1) was doubled (level 2) over the course of the treatment series. The figure indicates positive correlations between baseline heart rates and peak heart rates at both levels and the absence of higher peak heart rates when charge doses were doubled. Mean peak heart rate at level 1, 130 ± 19 bpm; mean peak heart rate at level 2, 123 ± 20 bpm.

We could not reproduce the ‘general absence of correlation between peak and baseline HRs,’ as stated by Swartz in 1999 [10]. In our sample of 119 patients, a correlation was present in RUL as well as in BF ECT, and the impact of baseline HR on peak HR remained significant after adjustment for covariates (Table 3). A positive correlation was equally evident in a second sample of 32 treatments in which the charge doses had been doubled (*r* = 0.515, *p* = 0.003, Figure 2). The lack of significance in the study of Swartz is probably due to the small number of patients (*n* = 24).

This small sample size also formed the statistical basis for the ‘Benchmark Method’ [11], in which the stimulus dose is to be set according to the peak HR obtained in previous seizures. The method requires (1) a causal relationship between charge dose and peak HR and (2) another linkage of higher peak HRs to greater treatment efficiency. Our study found no evidence of a positive correlation between stimulus dose and peak HR in RUL or BF ECT. The coefficient was also insignificant in regression analysis (*B* = −0.015, *p* = 0.250). There was no evidence that the administration of higher stimulus doses in the first treatment of a series caused higher heart rates.

We therefore investigated the relationship between stimulus dose and heart rate in an additional intraindividual comparison. This involved all patients where the initial stimulus doses had been doubled during their treatment series (*n* RUL = 16, *n* BF = 16). Subconvulsive stimuli were not taken into account. Although the mean charge dose in these 32 patients had been doubled from 253 ± 106 mC to 507 ± 211 mC, there was no corresponding increase in the peak HRs. On the contrary, their mean fell from 130 ± 19 bpm to 123 ± 20 bpm (*p* = 0.003, *t* test). This decrease is not surprising given that the baseline HRs fell from 90 ± 18 bpm in the first treatments to 83 ± 16 bpm in the subsequent treatments. Likewise, seizure durations fell from 45 ± 12 to 34 ± 11 seconds. Doubling the stimulus dose in the course of a treatment series seems to be a futile attempt to achieve higher peak HRs (Figure 2).

### Increase and decrease of heart rate

Heart rate during the stimulation period of ECT is generally expected to fall [12,13]. This study is the first to demonstrate an increase in heart rate during the bifrontal application of electrical stimuli with an inter-electrode distance ≤ 8 cm. The impact of electrode positioning on cardiac action has been documented repeatedly [9,12]; hence, the electrode distance seems crucial to the new finding. A possible physiological mechanism underlying this heart rate increase is discussed below.

In the present study, the time from the onset of stimulation until the heart rate started rising above baseline (*T*_rise_) served as an outcome variable. In a previous analysis of the same patient sample [9], we had chosen the longest interval between two heartbeats to quantify cardiac action during the stimulation phase. This cardiac pause length had varied greatly in the RUL group (1.21–11.20 s), and it had been impossible to identify any predictors. Taking ‘*T*_rise_’ as an outcome variable instead of the ‘stimulation interval’ yielded markedly better results. Age, baseline HR, and stimulus duration turned out to be significant predictors in the RUL group, explaining more than 50% of the variation, as shown in Table 3. The time span from the onset of stimulation until the heart rate starts to rise above baseline evidently reflects the interplay of parasympathetic and sympathetic forces during stimulation more precisely than the longest interval between two heartbeats.

The effect of stimulus duration on *T*_rise_ in the RUL group is in line with recently published findings. Stewart et al. [12] found a greater heart rate deceleration and more asystole events in longer stimulus trains, and Coughlin et al. [13] observed longer cardiac pauses when the stimulus durations were doubled from 2 to 4 s. Different outcome variables notwithstanding, all the studies agree that bradycardia or asystole are prolonged in the case of longer RUL stimulation.

Incidents of temporary asystole concomitant with the end of seizure signs have been described repeatedly [14-17]. In 1984, Larson et al. [2] assessed the utility of ECT-induced tachycardia as a measure of seizure length. They marked the sharp decline in heart rate as the end point of intraictal tachycardia. The point at which the heart rate graph crosses its baseline level again (*T*_base_) can often be determined more simply. This point, however, is reached more rapidly in the case of a high baseline or a low peak HR. *T*_dec_ is not affected and seems to be more appropriate for marking the onset of the post-ictal period of vagal dominance.

### Possible physiological mechanisms

The parasympathetic surge during stimulation depended on the positioning of the stimulus electrodes; it appeared with the first cardiac action starting in the stimulation period, and it was strongly linked to stimulus train duration. Bradycardia was also visible during irregular RUL stimulations. In the case of an interrupted treatment, we recorded a prompt heartbeat slowdown induced by no more than 14 pulses of 0.5 ms each (frequency, 40 Hz; train duration, 0.18 s). All these observations are explicable by assuming direct stimulation of the vagus nerve if the electrical field is placed in its proximity.

**Figure 3:**
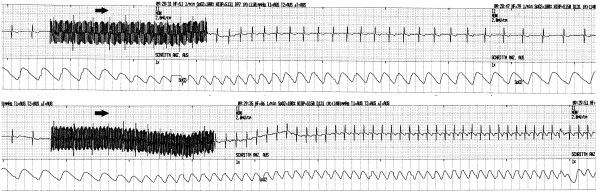
**Sympathetic surge during subconvulsive and convulsive bifrontal stimulation.** The figure shows ECT no. 4 in a 66-year-old male patient. Stimulus electrodes had been placed at a distance of 6 cm, that is, a distance of 10 cm between the midpoints. Arrows indicate the onset of tachycardia. The first stimulation (203 mC, 7.5 s, 30 Hz, 0.50 ms) was followed by neither motor nor EEG seizure signs. The subsequent stimulation (280 mC, 7.7 s, 40 Hz, 0.50 ms) led to a seizure of 60-s duration.

The quick activation of the sympathetic nervous system in the first half of the stimulation period was only traceable in the ECG records of BF treatments. As evident from its rapid onset, this tachycardia is neurally mediated. Epinephrine from the adrenal glands might contribute to cardiovascular action later on but cannot play a part at an early stage. A valuable insight into the physiological mechanisms was provided by subconvulsive stimulations, i.e., stimulations followed by neither motor nor EEG seizure signs. There were no such cases within the RUL sample and six (11%) in the BF group; all were successfully re-stimulated at higher doses. Cardiac action was similar in subconvulsive and convulsive stimulations, with five of the patients showing cardioacceleration during application of the electrical pulses. Subsequently, the heart rate fell below baseline level in three patients within 4.2–17.8 s following completion of the subconvulsive stimuli. The onset of tachycardia during BF stimulation can hence be attributed to the application of electrical pulses rather than to the seizure itself (Figure 3). Bifrontal electrical stimulation can activate the sympathetic nervous system for some seconds even in cases of a subconvulsive charge dose.

At the end of the seizures, some heart rate graphs gradually drifted towards baseline. In 78% of cases, however, they fell below that level and a steep, non-steady decline was recorded in 71%. These time curves are indicative of additional activation of the vagus nerve. Unlike the vagal surge during stimulation, it did not depend on electrode positioning. A potential mechanism is reflex bradycardia. Neurally mediated sympathetic activity is mainly based on norepinephrine. The IV infusion of this neurotransmitter rapidly leads to vasoconstriction and blood pressure elevation and is consistently followed by bradycardia. In keeping with neural mediation, arterial line recordings in ECT showed maximum systolic and diastolic blood pressure levels within 7 s of the first post-stimulus heartbeat [3]. The baroreceptor response is to be expected as a result.

## Conclusions

In this study, stimulus electrode positioning had a marked effect on cardiac action during electroconvulsive therapy in the stimulation phase. We did not find an effect on heart rate during the subsequent convulsive period. The influence of the patients' age on cardiac action in ECT seemed to be only of minor importance: bradycardia lasted longer during RUL stimulation; the heart rate reached its maximum a little later and had a tendency to decrease more gradually at the end of the seizure. The common decline of autonomic nervous system function in old age offers a simple explanation for this blunted heart rate response. The stimulus dose had no independent effect on heart rate, particularly peak heart rate, in fully developed seizures. In consequence, peak heart rate is an unsuitable parameter for adjusting the stimulus dose.

## Abbreviations

BF: Bifrontal; ECT: Electroconvulsive therapy; HR: Heart rate; RUL: Right unilateral; Tbase: Time until the heart rate falls to baseline level; Tdec: Time to the steepest heart rate decline during and after the seizure; Tmax: Time to peak heart rate; Trise: Time from the onset of stimulation until the heart rate rises above baseline level.

## Competing interests

The author declares that there are no competing interests.
